# Identification of potential metabolic biomarkers in predicting esophageal varices needing treatment in patients with liver cirrhosis

**DOI:** 10.1038/s41598-021-99198-9

**Published:** 2021-10-04

**Authors:** Chung-Man Moon, Yun-Young Lee, Suk-Hee Heo, Sang-Soo Shin, Yong-Yeon Jeong

**Affiliations:** 1 Research Institute of Medical Sciences, Chonnam National University, Gwangju, Republic of Korea; 2 Department of Radiology, Chonnam National University Hospital, Gwangju, Republic of Korea; 3 Department of Radiology, Chonnam National University Medical School, 42 Jebong-ro, Dong-gu, Gwang-ju, 61469 Republic of Korea; 4 Department of Radiology, Chonnam National University Hwasun Hospital, Hwasun, Republic of Korea

**Keywords:** Liver cirrhosis, Predictive markers

## Abstract

The goal of this study was to determine the diagnostic performance of in vivo quantitative proton magnetic resonance spectroscopy (^1^H-MRS) to identify the presence of esophageal varices needing treatment (VNT), as well as investigate its correlation with clinical characteristics in patients with liver cirrhosis. Forty cirrhotic patients without VNT showing the negative red color sign, and 40 cirrhotic patients with VNT showing positive red color sign underwent laboratory tests, esophago-gastro-duodenoscopy, and ^1^H-MRS with single-voxel localization in the cirrhotic liver parenchyma. The levels of lactate + triglyceride (TG) and choline in cirrhotic patients with VNT were significantly higher than those in cirrhotic patients without VNT. In multivariate analysis, spleen diameter, platelet count, and platelet count/spleen diameter ratio, as well as lactate + TG, and choline were associated with the presence of VNT. Moreover, lactate + TG and choline levels were positively correlated with spleen diameter and negatively correlated with platelet count in the combined group of cirrhotic patients with and without VNT. Our study demonstrated that higher hepatic lactate + TG and choline levels in cirrhotic patients in conjunction with longer spleen diameter, lower platelet counts, and lower ratios of platelet count to spleen diameter were associated with the presence of esophageal VNT and the risk of developing variceal bleeding. Therefore, in vivo ^1^H-MRS might be an effective tool for diagnosing and predicting esophageal VNT in patients with liver cirrhosis.

## Introduction

Liver cirrhosis is a severe and irreversible disease of the liver that is known to result in metabolic hepatic failure and portal hypertension^1^. One of the major complications of portal hypertension is the development of esophageal varices (EVs), which are correlated with the severity of chronic liver disease^2^. Although screening endoscopy for esophageal varices needing treatment (VNT) has been recommended for cirrhotic patients due to the high mortality rate associated with variceal bleeding^3^, this method is invasive and frequently requires sedation, limiting its clinical utility as a screening test. Therefore, there is a clinical demand for noninvasive assessment, particularly in predicting active bleeding from EV.

In this context, there has been an increasing interest in discovering noninvasive parameters that might help detect the presence of EV or determine groups at high risk of developing variceal bleeding among cirrhotic patients, such as the diameter of the spleen or portal vein, platelet count, Child–Pugh score, prothrombin time assay, or a combination of measurements with multiple biomarkers, along with an ultrasound or magnetic resonance elastography^4^. However, consistent utilization of these methods might not be supported when applied to other independent patient series. Among non-invasive methods, two-dimensional shear wave elastography based on shear waves embedded in diagnostic ultrasound systems has emerged as one of the newest diagnostic tools that could be particularly useful for predicting high-risk varices in patients with compensated advanced chronic liver disease^5,6^. Moreover, as a robust noninvasive in vivo molecular imaging technique, proton magnetic resonance spectroscopy (^1^H-MRS) facilitates the identification of metabolite profiles in vivo, providing a biochemical characterization of normal and abnormal tissues through quantitative measures of cellular metabolites^7^. The liver is one of the most metabolically diverse organs, as it is involved in many critical metabolic processes^8^. Clinically, metabolic information indicative of liver disease could help clinicians better characterize disease pathophysiology. Although cirrhosis represents a major change in the tissue, conventional approaches for assessing this disease rely on relatively few biomarkers and are not sufficient when providing global biochemical properties and definitive diagnoses^9^. Based on clinical observations, we hypothesized that quantitative ^1^H-MRS for hepatic metabolite changes in vivo might serve as a potential tool in diagnosing and predicting esophageal VNT in cirrhotic patients.

Thus, the purpose of our study was to evaluate the diagnostic performance of ^1^H-MRS and investigate its correlation with clinical characteristics in cirrhotic patients with and without VNT with conventional endoscopy as the reference standard.

## Results

### Comparison of clinical characteristics

Table 1 presents the characteristics of the two patient groups. Cirrhotic patients with VNT had lower platelet counts, lower levels of serum aspartate aminotransferase (AST) and alanine aminotransferase (ALT), higher total bilirubin, lower serum albumin levels, longer spleen diameters, and lower ratios of platelet count to spleen diameter than those without VNT. Except for AST and ALT levels, these clinical characteristics were all significantly different between the two groups (*P* < 0.05). When the study population was stratified according to the spleen diameter and platelet count, the cirrhotic patients with VNT showed a stepwise manner increase of spleen diameter with reduction of platelet count compared to the cirrhotic patients without VNT.

**Table 1:** Characteristics of cirrhotic patients with and without esophageal varices needing treatment (VNT).

Variables	Patients without VNT (n = 40)	Patients with VNT (n = 40)	P-value
Mean age (y)	59.00 ± 21.45	63.00 ± 22.90	0.02
No. of men (%)	20 (50.0)	20 (50.0)	–
Aspartate aminotransferase (U/L)	61.89 ± 40.12	40.00 ± 10.96	0.23
Alanine aminotransferase (U/L)	55.00 ± 35.41	21.67 ± 6.25	0.48
Total bilirubin (mg/dL)	1.17 ± 0.43	1.39 ± 0.44	0.00
Albumin (g/dL)	4.13 ± 1.51	3.30 ± 1.01	0.00
Spleen diameter (cm)	11.08 ± 1.24	13.24 ± 1.99	0.00
Platelet count (× 1000/µL)	183.43 ± 84.24	103.33 ± 38.58	0.00
Platelet count/spleen diameter ratio	16.98 ± 8.84	8.14 ± 3.83	0.00

Independent two-sample *t*-test was used for statistical analysis.

### Comparison of the hepatic metabolite levels for lactate + TG, choline, and TG

**Figure 1:**
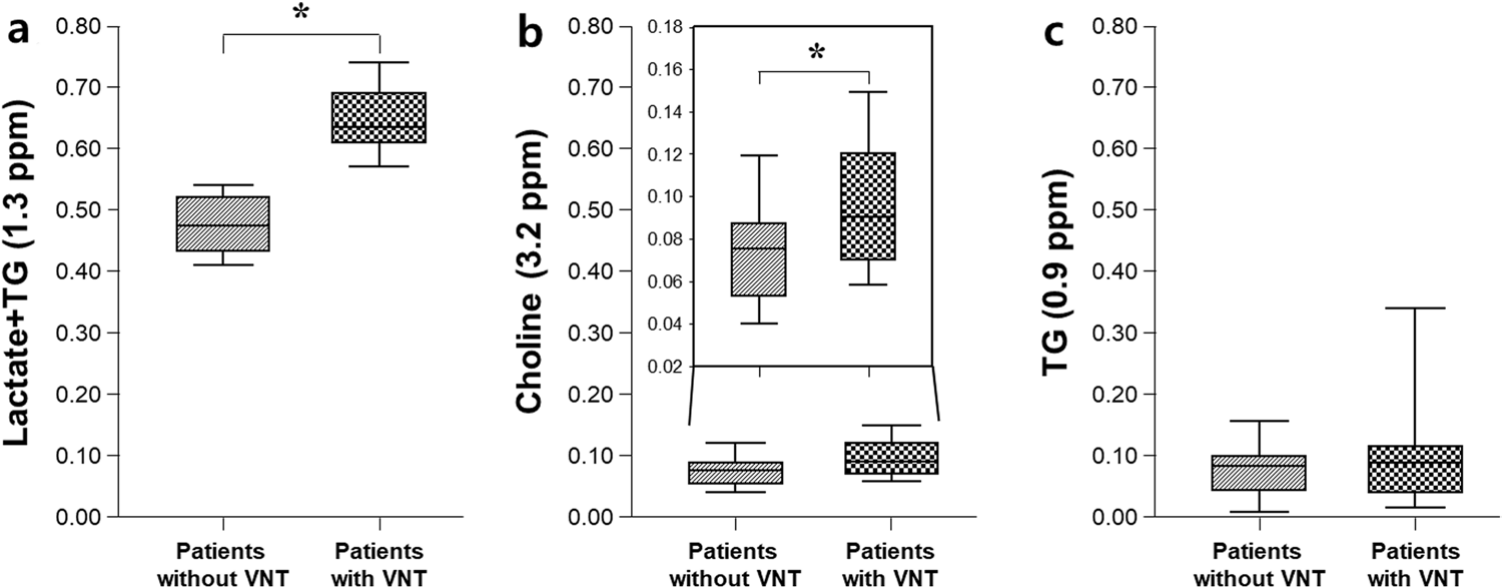
Quantitative comparison of the cellular metabolites for (**a**) lactate + TG (1.3 ppm), (**b**) choline (3.2 ppm), and (**c**) TG (0.9 ppm) in cirrhotic patients with and without VNT. The differential metabolite levels between the two groups were analyzed using an analysis of covariance (ANCOVA) with adjustments for age and sex at *P* < 0.05. *Significant difference between cirrhotic patients with and without VNT. TG, triglyceride; VNT, varices needing treatment.

Cellular metabolites showed distinct patterns of hepatic metabolism between the groups (Fig. 1). The levels of lactate + TG and choline in cirrhotic patients with VNT were significantly higher compared to those in cirrhotic patients without VNT (*P* < 0.05), while the level of TG at 0.9 ppm was similar between the two patient groups.

The intraclass correlation coefficient (ICC) was 0.949 (95% CI, 0.921 – 0.967; *P* < 0.001) for lactate + TG, 0.869 (95% CI, 0.795 – 0.916; *P* < 0.001) for choline, and 0.958 (95% CI, 0.934 – 0.973; *P* < 0.001) for TG.

### Independent predictive factors for the presence of VNT in cirrhotic liver

The association of clinical characteristics and metabolite concentrations by ^1^H-MRS with the presence of VNT was shown in Table 2. In univariate analysis, total bilirubin, albumin, spleen diameter, platelet count, platelet count/spleen diameter ratio, lactate + TG, and choline were independent predictors for the presence of VNT (*P* < 0.05) except AST (*P* = 0.232), ALT (*P* = 0.483), and TG (*P* = 0.908).

**Table 2:** Regression analysis for verifying the association of various variables with the presence of VNT.

Variables	Univariate analysis		Multivariate analysis	
	β coefficient	P-value	β coefficient	P-value
Aspartate aminotransferase (U/L)	0.135	0.232	–	–
Alanine aminotransferase (U/L)	−0.080	0.483	–	–
Total bilirubin (mg/dL)	**0.432**	**0.000**	0.044	0.355
Albumin (g/dL)	−**0.392**	**0.000**	−0.021	0.637
Spleen diameter (cm)	**0.552**	**0.000**	**0.433**	**0.001**
Platelet count (× 1000/µL)	−**0.526**	**0.000**	−**0.653**	**0.013**
Platelet count/spleen diameter ratio	−**0.549**	**0.000**	−**0.607**	**0.035**
Lactate + TG	**0.821**	**0.000**	**1.451**	**0.000**
Choline	**0.340**	**0.002**	**0.299**	**0.000**
TG	−0.013	0.908	–	–

Bolded values are significant at *P* < 0.05.

VNT, varices needing treatment; TG, triglycerides.

In a multivariate analysis that was performed using the variables showing significant P-value in univariate analysis, spleen diameter, platelet count, platelet count/spleen diameter ratio, lactate + TG, and choline were independently associated with the presence of VNT (*P* < 0.05) except total bilirubin (*P* = 0.355), and albumin (*P* = 0.637).

### Correlation of the hepatic metabolite levels with spleen diameter and platelet count

**Figure 2:**
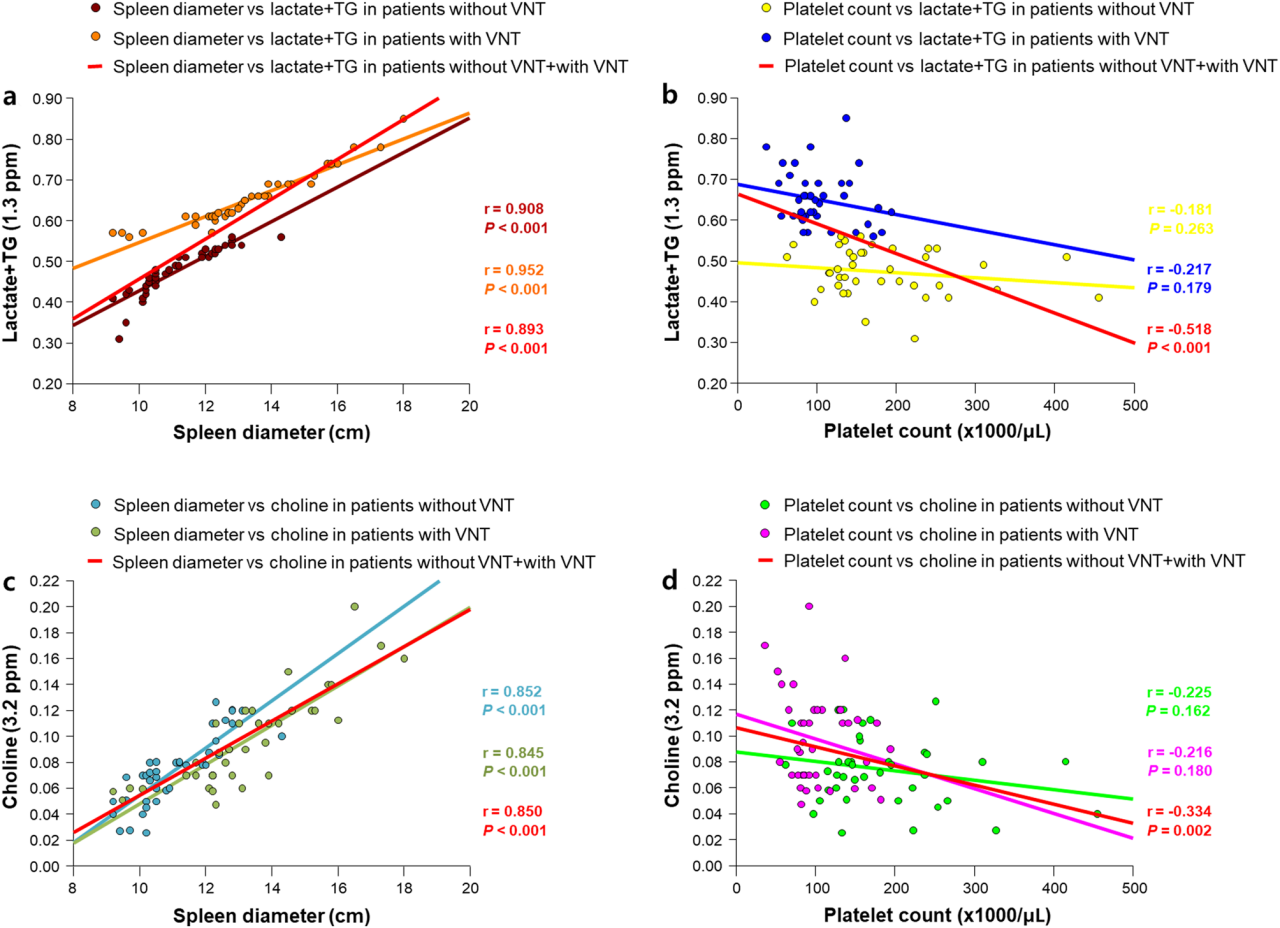
Correlations of lactate + TG and choline metabolites with spleen diameter and platelet count in cirrhotic patients with and without VNT, and in the combined group of cirrhotic patients without/with VNT.

Pearson’s correlation coefficients were calculated to evaluate the relationship between the clinical measurements and quantitative concentrations of ^1^H-MRS metabolites. Spleen diameter showed a significantly positive correlation with lactate + TG and choline in cirrhotic patients without VNT (lactate + TG: r = 0.908, *P* < 0.001; choline: r = 0.852, *P* < 0.001) and with VNT (lactate + TG: r = 0.952, *P* < 0.001; choline: r = 0.845, *P* < 0.001), as well as in the combined group of cirrhotic patients without/with VNT (lactate + TG: r = 0.893, *P* < 0.001; choline: r = 0.850, *P* < 0.001) (Fig. 2a,c). While platelet count was significantly negatively correlated with lactate + TG and choline in the combined group of cirrhotic patients without/with VNT (lactate + TG: r = -0.518, *P* < 0.001; choline: r = -0.334, *P* = 0.002), this negative correlation was not significant in cirrhotic patients without VNT (lactate + TG: r = -0.181, *P* = 0.263; choline: r = -0.225, *P* = 0.161) and with VNT (lactate + TG: r = -0.217, *P* = 0.179; choline: r = -0.216, *P* = 0.180) (Fig. 2b,d).

## Discussion

The current study was the first to identify metabolic biomarkers that could be used to predict esophageal VNT in cirrhotic patients using ^1^H-MRS in conjunction with their relationships with the clinical characteristics of spleen diameter and platelet count. Through both univariate and multivariate regression analysis, we found that hepatic lactate + TG and choline measured using ^1^H-MRS were independently associated with the presence of VNT in cirrhotic patients, which could be used as potential risk factors together with clinical characteristics of spleen diameter, platelet count, and platelet count/spleen diameter ratio. Additionally, in correlation analysis, the ^1^H-MR spectroscopic signal intensities of both lactate + TG and choline were significantly associated with spleen diameter and platelet count in cirrhotic patients with/without VNT.

In order to successfully obtain MR spectra and increase the accuracy of hepatic metabolite concentrations measured using in vivo ^1^H-MRS, the respiratory movement was minimized with the use of a breath-hold sequence. Indeed, in our study, the ICC values as a reproducibility index of in vivo ^1^H-MRS were above 0.85 indicating a good agreement. Therefore, it is anticipated that the values of in vivo ^1^H-MRS as a diagnostic tool might be promising to evaluate possible esophageal variceal bleeding as well as provide valuable information for understanding the cellular alterations in hepatic metabolism related to progressive EV in cirrhotic liver disease.

As an interesting feature of hepatic metabolite differences, the lactate + TG level was significantly higher in cirrhotic patients with VNT than in cirrhotic patients without VNT. Although the lactate signal at 1.3 ppm overlapped with large TG resonance in the liver and could not be separated from TG peak in our spectra^10^, we assumed that lactate contributed to differential patterns of metabolite changes between cirrhotic liver with and without VNT, similar to previous studies^11,12^ on in vivo ^1^H-MRS with a long TE, which demonstrated a disease-specific lactate + TG signal intensity. The liver plays an important role in modulating cellular homeostatic pathways such as the metabolism of organic acid anions including lactate and amino acids^13,14^. However, in chronic liver diseases, lactate clearance is impaired due to a reduction in functional hepatocytes, while healthy liver has a major functional reservoir of metabolizing lactate^13,14^. Several studies^13,15,16^ using hyperpolarized ^13^C-MRS with liver disease models reported that the lactate-to-pyruvate ratio could be used as a metabolic marker indicating an inadequate supply of oxygen and glucose, suggesting that higher lactate levels compared with normal controls are induced by hypoxic and hypermetabolic states under anaerobic glycolysis in pathological states. More importantly, our study demonstrated that hepatic metabolite values for lactate + TG were associated with the presence of esophageal VNT. However, the relationships between the metabolic parameters and the endoscopic findings of EVs have been poorly investigated. A recent study^17^ reported that the presence of EVs and the risk of developing variceal bleeding correlated with the severity of cirrhosis. Enomoto et al.^18^ reported the association of the branched-chain amino acids to tyrosine ratio (BTR) with the severity of liver fibrosis and EVs in patients with hepatitis C virus-positive chronic liver disease. In that study, a decreased value of BTR was found to be associated with the progression of liver fibrosis and severity of varices.

In our study, when evaluating the association of spleen diameter and platelet count with metabolite alterations in cirrhotic patients, spleen diameter was positively correlated with lactate + TG level in both cirrhotic patients without and with VNT, as well as in the combined group of cirrhotic patients without/with VNT. These positive correlations indicate that any increase in spleen diameter in cirrhotic patients was associated with higher levels of hepatic metabolites, and therefore with impaired metabolic status associated with the progression of EV as well as the presence of esophageal VNT. Additionally, in the combined group of cirrhotic patients without/with VNT, platelet count was negatively correlated with lactate + TG level, suggesting that higher levels of hepatic metabolites could be related to the severity of impaired metabolic status, which might be associated with the development of esophageal VNT in patients with cirrhotic liver. Therefore, the increased lactate + TG level might be a noninvasive metabolic biomarker reflecting disease-specific metabolism in the hepatic pathophysiology to predict the occurrence of esophageal VNT or help determine an effective follow-up strategy.

Our results also revealed that in cirrhotic patients with VNT, the choline metabolite levels were significantly higher than those in cirrhotic patients without VNT. Choline is an important constituent of the cell membrane in phospholipid metabolism and is an active marker of cellular proliferation, indicating that an elevated choline peak may be associated with increased membrane phospholipid biosynthesis^19^. Given the altered level of choline-containing compounds including choline, phosphocholine, glycerophosphocholine, and taurine in cirrhotic liver, a previous study^9^ reported that alterations of choline-containing compounds may suggest abnormal synthesis or degradation of cell membranes, or may just be related to the mobility of the choline-containing compound parts. In our correlation analysis, choline levels were positively correlated with spleen diameter in both cirrhotic patients without and with VNT, as well as in the combined group of cirrhotic patients without/with VNT. Moreover, these levels were negatively correlated with platelet count in the combined group of cirrhotic patients without/with VNT. These findings may suggest that in cirrhotic liver at the risk of developing variceal bleeding, the increase in choline level as a cellular biomarker reflects the ongoing destruction of functional hepatic tissue and hepatocytes, which could eventually result in hepatic failure.

Our study has several limitations. First, we did not apply multi-voxel scanning when acquiring in vivo quantitative ^1^H-MRS because such a technique requires much longer scanning and breath-holding times. Second, the number of patients in our study was relatively small. Further, in our study, a validation cohort was not introduced. Therefore, a larger number of patients in a prospective study might be necessary to verify the performance of the metabolic biomarkers tested in our study in an independent clinical cohort. Third, regarding the etiologies of liver cirrhosis, 96.3% of the patients in our study were viral hepatitis-induced cirrhotic patients. Further studies are necessary to perform on cohorts with other etiologies such as NAFLD/NASH and alcohol. Fourth, as mentioned in the discussion, the lactate signal at 1.3 ppm could not be separated from the TG peak. Thus, utilizing high-field magnetic resonance imaging (MRI) equipment, spectrally overlapped lactate and TG signals at 1.3 ppm should be separated and quantified in future studies.

In conclusion, we utilized ^1^H-MRS to quantify hepatic lactate + TG and choline levels in cirrhotic patients, demonstrating that higher values of these metabolites were associated with the presence of esophageal VNT and the risk of developing variceal bleeding. Further, those metabolic changes were correlated with longer spleen diameter and lower platelet count.

## Methods

### Patients

This prospective study was approved by the institutional review board of Chonnam National University Hospital and conformed to the ethical guidelines of the 2008 Declaration of Helsinki. All subjects provided written informed consent. To quantify significant differences in cellular metabolite levels between the groups with a level of significance of α = 0.05 and β = 0.2 in this study, at least 40 subjects had to be included per group.

Between November 2017 and December 2020, 517 patients with cirrhosis confirmed by liver biopsy or imaging findings, such as morphologic changes in the liver or sequelae of portal hypertension^20^, were referred to the department of radiology, where a hepatic ^1^H-MRS was performed as part of routine imaging. Among them, patients who met the following criteria were excluded based on our protocol: the presence of multiple or infiltrative hepatocellular carcinoma (HCC) with limited parenchymal evaluation (n = 155); fatty deposition (more than 5%) in the liver (n = 182); presence of a lipiodol-uptake lesion in the liver due to previous trans-arterial chemoembolization for HCC (n = 41); patients who did not undergo MRI examination due to poor respiratory condition (n = 25); patients who had not undergone esophago-gastro-duodenoscopy (EGD) within the previous 6 months (n = 19); and patients with a history of treatment for EV (n = 15).

An EGD was performed by gastroenterologists who had more than 5 years of experience in clinical practice. EVs were classified according to location, form, color, and the red color sign as follows^21^: (1) the location of the varices was classified into the upper, middle, or lower third of the esophagus or upper stomach; (2) variceal forms included no observation of varices (F0), small and straight varices (F1), enlarged and tortuous varices (F2), or large and coil-shaped varices (F3); (3) the color of the varices was graded as white (Cw) or blue (Cb); and (4) the red color sign was considered to be present in cases with dilated, small vessels (red wale sign), and telangiectasias or cherry-red spots on the surface of the varices. Cirrhotic patients were divided into two groups. One group included patients without VNT (F0 or F1) showing a negative red color sign (no history of bleeding) and the other group included patients with VNT (F2 or F3) showing the presence of esophageal variceal bleeding (presence of red color signs, which are venules or red spots on the varices)^2^.

Consequently, this prospective study consisted of 40 cirrhotic patients without VNT including negative red color signs and 40 cirrhotic patients with VNT including positive red color signs. The causes of liver cirrhosis were hepatitis B (n = 51), hepatitis C (n = 17), both hepatitis B and C (n = 9), alcohol‐related (n = 2), and idiopathic (n = 1).

### MR imaging and spectroscopy

**Figure 3:**
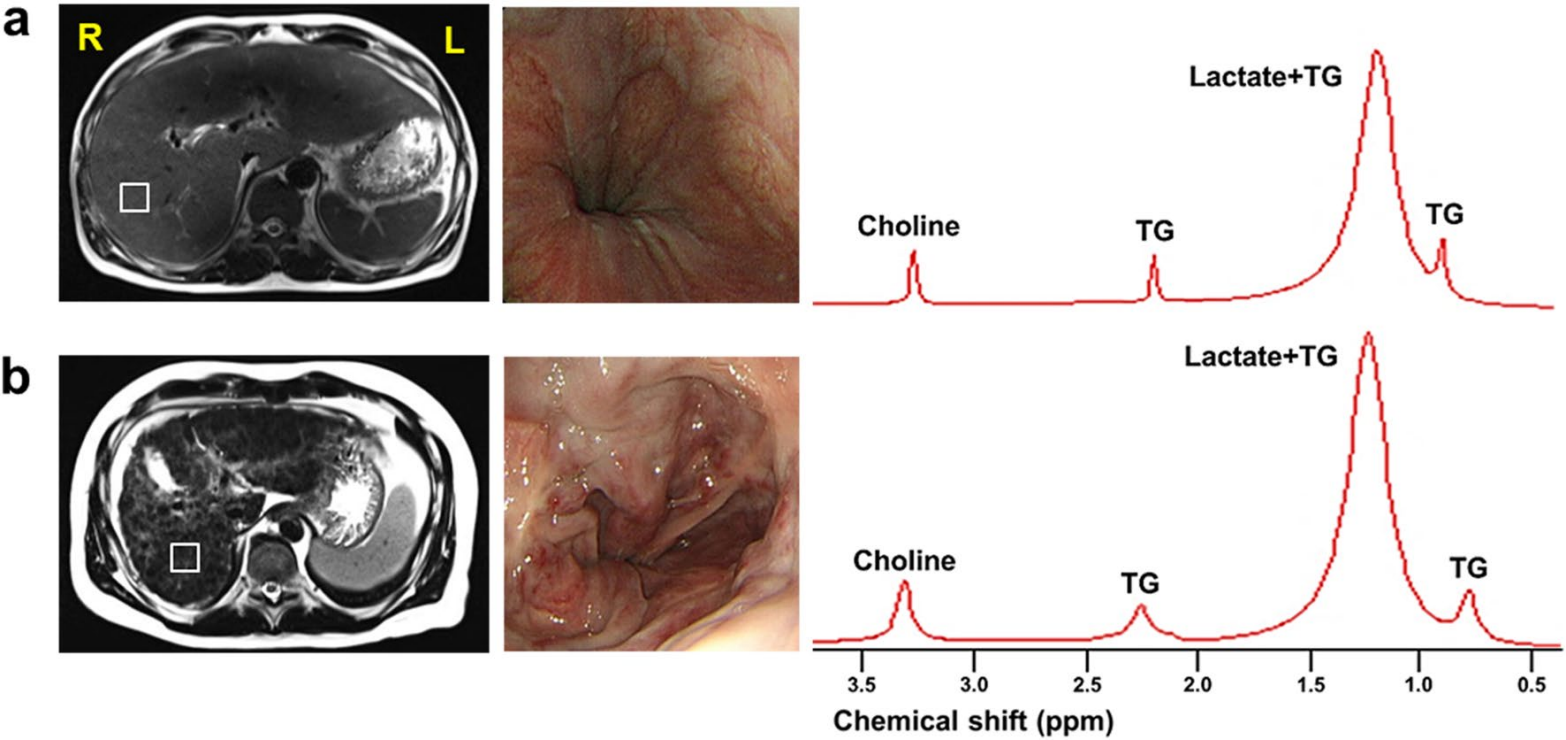
Axial MRI (left) with endoscopic image (middle) and representative MR spectra (right) acquired from a voxel (white square on axial MR image) localized in the right lobe of the liver in a cirrhotic patient without VNT (**a**), and a cirrhotic patient with VNT (**b**). L, left; R, right; TG, triglycerides; VNT, varices needing treatment.

MR images and spectra were acquired using a 3-T MR scanner (Magnetom TimTrio, Siemens Healthcare, Erlangen, Germany). The T2-weighted images were acquired using a half-Fourier acquisition single-shot turbo spin-echo (HASTE) sequence with the following parameters: repetition time/echo time (TR/TE) = 2000/167 ms; field of view = 380 × 380 mm; matrix size = 320 × 256; number of excitations = 1; slice thickness = 5 mm; interslice gap = 0.5 mm; number of slices = 32 ~ 50; and scan time = 60–90 s. To saturate fat and detect lactate and choline metabolites with a long T2 relaxation time, ^1^H single‐voxel spectra was acquired using a point-resolved spectroscopy sequence with the following parameters: TR/TE = 2000/288 ms, six acquisitions (within a single breath-hold), 2000 Hz spectral width, and 2 × 2 × 2 cm^3^ voxel size, which was in the region of interest (ROI) voxel on the cirrhotic liver parenchyma^11,12,22^. Figure 3 shows representative localized voxel and ^1^H-MR spectra with a long TE acquired from each group. MRS acquisition began when the level of water suppression was over 90% and the bandwidth was below 10 Hz after auto-shimming. To reduce respiratory motion artifacts, a compression belt was used while the patients’ breathing was monitored, and MRS acquisition was stopped earlier or whenever the patient had to breathe again^11^.

### MR spectra analysis

MR spectra were analyzed using a Java-based MR user interface software (jMRUI version 4.0; developed by A. van den Boogaart, Katholieke Universiteit Leuven, Leuven, Belgium) following the procedure described in previous studies^11,12^. Major hepatic metabolites were assigned from the MR spectra as follows: triglyceride (TG, 0.9 ppm), lactate + TG (1.3 ppm), and choline (3.2 ppm). All spectra were then fitted in the time domain using a non-linear least-squares algorithm in the jMRUI software package (AMARES). The signal intensity of all spectra was normalized to that of the residual water peak at 4.7 ppm as an internal reference.

### Clinical measurements

To evaluate blood cell counts and liver function as blood‐based biomarkers, blood samples were analyzed using a routine clinical chemistry analyzer. The spleen diameter was measured on MR images and was defined as the greatest longitudinal dimension at the level of the splenic hilum on the PACS monitor using electronic calipers^23^. Spleen diameter and platelet count have been proposed as predictive values that may be directly or indirectly associated with the presence of EV, in particular, the platelet count/spleen diameter ratio is mostly used as the noninvasive predictor of EV^24^.

### Statistical analysis

Data were statistically evaluated using independent two-sample t-tests to compare clinical characteristics and analysis of covariance (ANCOVA) with adjustments for age and sex to compare the metabolic levels using statistical software (SPSS for Windows, version 18; SPSS Inc, Chicago, IL) between the two groups. The ICC as a reproducibility index was used to assess the reproducibility of quantitative measurements of ^1^H-MRS metabolites. Additionally, univariate and multivariate regression analysis were used to verify the association of clinical characteristics and in vivo metabolites with the presence of VNT. The correlation of metabolite levels with splenic diameter, and platelet count was assessed using Pearson’s correlation.

## Data Availability

The data that support the findings of this study are available from the corresponding author upon reasonable request.
